# Bis[1,2-bis­(4-*tert*-butyl­phen­yl)ethyl­ene-1,2-di­thiol­ato(1−)]nickel(II) pentane 0.25-solvate

**DOI:** 10.1107/S205698902300097X

**Published:** 2023-02-17

**Authors:** Titir Das Gupta, Jack Applebaum, William Broussard, Carson Mack, Che Wu, Joel T. Mague, James P. Donahue

**Affiliations:** a Department of Chemistry, Tulane University, 6400 Freret Street, New Orleans, Louisiana 70118-5698, USA; Texas A & M University, USA

**Keywords:** crystal structure, di­thiol­ene, nickel, electron-withdrawing, electron-donating

## Abstract

The title compound, [Ni(C_22_H_26_S_2_)_2_], **1**, is a square-planar *D*_2*h*_-symmetric compound that occurs on a general position in non-centrosymmetric tetra­gonal *P*4_1_2_1_2 (No. 92) with ¼ eq of *n*-pentane (C_5_H_12_) as co-crystallite. Intra­ligand bond lengths show the di­thiol­ene ligands to be in their half-oxidized radical monoanionic form. Inter­molecular ^*t*^Bu-C—H⋯arene_centroid_ and ^*t*^Bu-C—H⋯NiS_2_C_2 centroid_ close contacts guide the packing arrangement for **1**.

## Abstract 2

Square-planar bis­[1,2-bis­(4-*tert*-butyl­phen­yl)ethyl­ene-1,2-di­thiol­ato(1–)]nickel(II) crystallizes on a general position in chiral *P*4_1_2_1_2, where asymmetry is induced by twisting of the aryl rings due to inter­molecular ^*t*^Bu-C—H⋯ring_centroid_ inter­actions in the packing pattern.

## 1. Chemical context

Group 10 metallodi­thiol­ene complexes have elicited considerable and sustained inter­est because their optical and solid-state properties are well suited for such important applications as reversibly bleaching dyes in neodymium YAG lasers (Mueller-Westerhoff *et al.*, 1991 ▸), as robust dyes for optical data storage (Nakazumi *et al.*, 1992 ▸), as non-linear optical devices (Deplano *et al.*, 2010 ▸) and as conducting (Robertson & Cronin, 2002 ▸; Kato, 2004 ▸; Ouahab, 1998 ▸) or magnetic materials (Robertson & Cronin, 2002 ▸; Ouahab, 1998 ▸; Faulmann & Cassoux, 2003 ▸). Among the ligand type generally, those with aryl (Ar) substituents enjoy the advantages of straightforward synthesis from readily accessible benzoin or benzil precursors and of qualitatively predictable effect upon redox potentials and absorption spectra. Our own inter­est in complexes featuring such ligands has been motivated by their potential to host, by means of appropriately set di­thiol­ene radicals, coherent quantum states for application in quantum computing and data storage (McGuire *et al.*, 2018 ▸). With the aim of broadening the window of redox potentials for the [Ar_2_C_2_S_2_^2–^] − e^−^ → [Ar_2_C_2_S**^.^** S^−^] oxidation, thereby creating the possibility for completely resolving and separately observ­ing these oxidation processes in mixed di­thiol­ene complexes of the form [(Ar_2_C_2_S_2_)*M*(tpbz)*M*(S_2_C_2_Ar’_2_)] (tpbz = 1,2,4,5-tetra­kis­(di­phenyl­phosphino)benzene; Ar ≠ Ar’), we have undertaken the synthesis and electrochemical characterization of a variety of [Ni(S_2_C_2_Ar_2_)_2_] complexes with either electron-withdrawing or electron-donating ring sub­stituents. In the course of this effort, crystalline samples of [Ni(S_2_C_2_(C_6_H_4_-4-^*t*^Bu)_2_)_2_] that were suited for crystallography were obtained. Herein, the details of this structure are described.

The 4,4′-di-*tert*-butyl­benzoin that serves as a di­thiol­ene ligand precursor is prepared from the corresponding benz­alde­hyde by a 1,4-dimethyl-1,2,4-triazolium iodide-mediated coupling reaction (Myles *et al.*, 2013 ▸). Following a procedure originally disclosed by Schrauzer (Schrauzer & Mayweg, 1965*a* ▸) and well vetted by others, benzoins and benzils are subject equally well to transformation to di­thiol­ene thio­phosphoryl sulfides (Schrauzer & Mayweg, 1965*b* ▸; Arumugam *et al.*, 2007 ▸) upon treatment with P_4_S_10_ in refluxing dioxane. Without the necessity of their being isolated and purified, the introduction of a Ni^2+^ salt to these di­thiol­ene thio­phosphoryl inter­mediates leads to the metal bis­(di­thiol­ene) complex as a charge-neutral species that precipitates from the reaction mixture. Execution of Schrauzer’s protocol using 4,4′-di-*tert*-butyl­benzoin produces [Ni(S_2_C_2_(C_6_H_4_-4-^*t*^Bu)_2_)_2_], **1**, in a yield of 32%. The bis­(4-*tert*-butyl­phen­yl)-substituted di­thiol­ene ligand has been used in the preparation and structural characterization of homoleptic Au^3+^ (Kokatam *et al.*, 2007 ▸), Pd^2+^ (Kokatam *et al.*, 2007 ▸), and Pt^2+^ complexes (Pap, *et al.*, 2007 ▸), but its Ni^2+^ compound, although investigated spectroscopically (Men *et al.*, 2008 ▸), has not been the subject of a crystallographic study. 
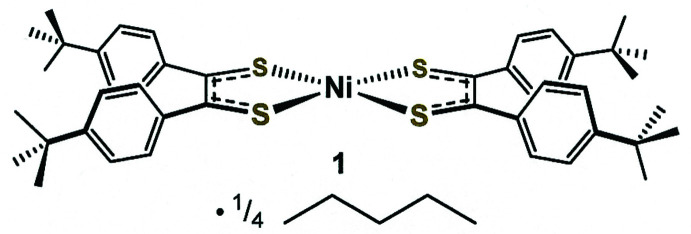


## 2. Structural commentary

**Figure 1:**
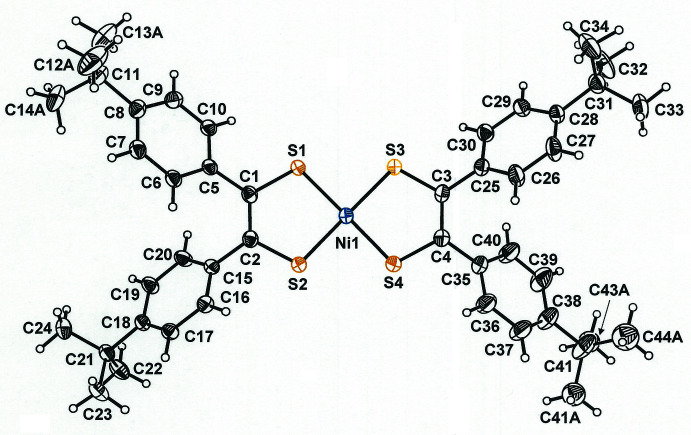
Atom labeling for **1**. Displacement ellipsoids are shown at the 50% probability level. For clarity, the disordered ^*t*^Bu groups (C11→C14*A* and C41→C44*A*) are edited to show only one of the two orientations.

**Figure 2:**
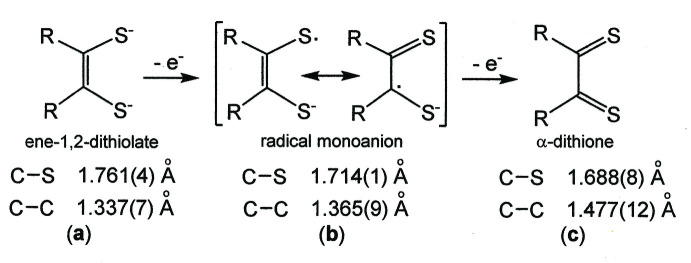
Redox levels of the di­thiol­ene ligand with typical intra­ligand bond lengths.

Compound **1** (Fig. 1 ▸) crystallizes in the non-centrosymmetric tetra­gonal space group *P*4_1_2_1_2 (No. 92) with ¼ eq of *n*-pentane (C_5_H_12_) and features a *c* axis much longer [65.014 (4) Å] than its other cell dimensions [11.7187 (4) Å]. The intra­ligand bond lengths (S—C ≃ 1.71 Å, C—C_chelate_ ≃ 1.37 Å) are indicative of the radical monoanionic redox state for the di­thiol­ene ligand [Fig. 2 ▸(*b*)]. The bond lengths presented in Fig. 2 ▸ are taken from well-defined nickel bis­(di­thiol­ene) complexes in which both ligands are fully reduced (Lim *et al.*, 2001 ▸), half-oxidized (Lim *et al.*, 2001 ▸), and fully oxidized (Bigoli *et al.*, 2001 ▸). The angles at which the arene rings meet the central NiS_4_C_4_ mean plane range quite narrowly [41.7 (1)–53.5 (1)°].

## 3. Supra­molecular features

**Figure 3:**
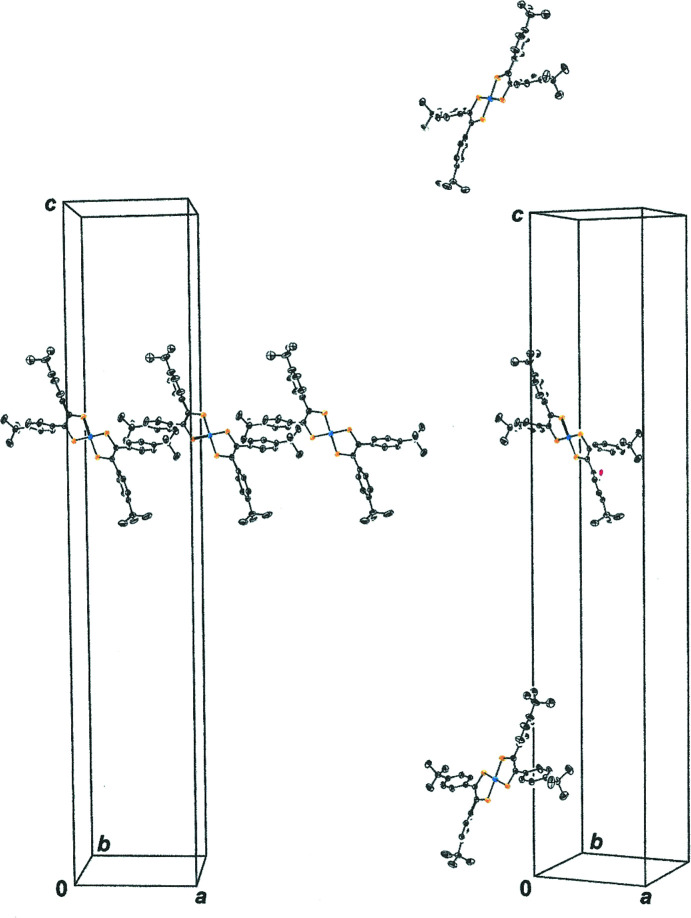
Mol­ecules of **1** related by translations along the *a* axis (left side). Mol­ecules of **1** related by the 2_1_ screw axis operation along *c* (right side). Displacement ellipsoids are drawn at the 50% level, and all H atoms are omitted for clarity.

**Figure 4:**
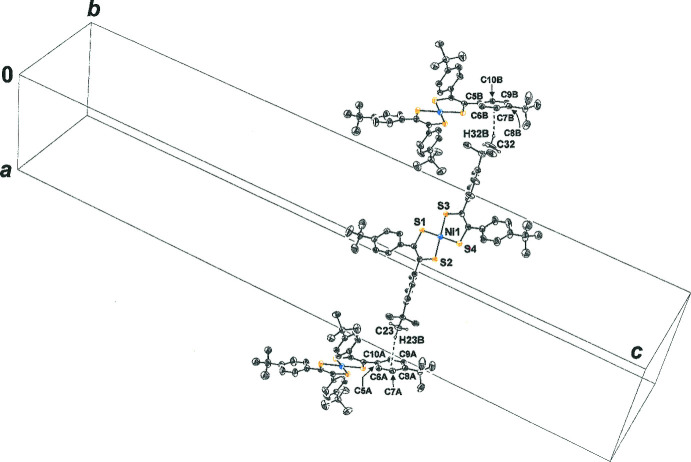
Inter­molecular arene_centroid_⋯H–C ^*t*^Bu inter­actions, shown as dashed lines, that guide the packing arrangement for **1**. The H23*B*⋯C5→C10_centroid_ and H32*B*⋯C5→C10_centroid_ contacts are 3.00 and 2.90 Å, respectively. Symmetry transformation used to generate equivalent mol­ecules: 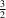 − *x*, − 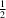 + *y*, 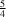 − *z*; − 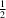 − *x*, 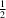 + *y*, 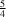 − *z*.

For **1**, the appreciably longer mol­ecular axis that bis­ects the di­thiol­ene C—C_chelate_ bonds and the non-planarity/non-orthogonality of the arene rings relative to the NiS_4_C_4_ core are features that support the occurrence of *P*4_1_2_1_2, as seen with similarly elongated mol­ecules bearing a twisted character [*cf.*, for example, ACAGAN (Dowd & Stevens, 2004 ▸); BALWAO (Trzeciak-Karlikowska *et al.*, 2011 ▸); CANCIH (Lin *et al.*, 2021 ▸)]. Simple translations relate one mol­ecule of **1** to another along the *a-* and *b*-axis directions (Fig. 3 ▸, left), while in the direction of the *c* axis, replication of **1** arises by movement along 2_1_ axes that are coincident with the *c* edges of the cell (Fig. 3 ▸, right) and by 4_1_ axes positioned parallel to the *c* axis at the middle of the *ac* and *bc* faces. Multiple inter­molecular ^*t*^Bu-C—H⋯arene_centroid_ and ^*t*^Bu-C—H⋯NiS_2_C_2centroid_ close contacts appear to play a decisive role in determining the packing symmetry patterns (Fig. 4 ▸). The most important of these inter­actions, as gauged by physical proximity, is the C22—H22*A*⋯Ni2S3S4C3C4_centroid_ contact (2.78 Å).

## 4. Database survey

Table 1 ▸ summarizes selected data pertinent to a set of structurally characterized Group 10 and 11 bis­(di­thiol­ene) complexes that are symmetrically substituted with the same arene rings, which now includes three complete series for Group 10 (Ar = Ph, MeO-4-C_6_H_4_, ^*t*^Bu-4-C_6_H_4_). The database entries included in this tabular survey are NIDPDS01 (Megnamisi-Belombe & Nuber, 1989 ▸), NIDPDS03 (Miao *et al.*, 2011 ▸), GOLRAA (Sheu & Lee, 1999 ▸), BUGDUC (Dessy *et al.*, 1982 ▸), SICWOR (Arumugam *et al.*, 2007 ▸), SONPUI (Chandrasekaran *et al.*, 2014 ▸), SOPMOB (Chandrasekaran *et al.*, 2014 ▸), ECEKAA (Miao *et al.*, 2011 ▸), DATTUR (Koehne *et al.*, 2022 ▸), JUHJUR (Nakazumi *et al.*, 1992 ▸), TEYSEW (Kokatam *et al.*, 2007 ▸), TIDBEO (Pap *et al.*, 2007 ▸), and TEYSAS (Kokatam *et al.*, 2007 ▸). Constancy of crystal system, space group, and unit-cell dimensions is found only for the Ar = Ph series, primarily owing to the absence *versus* presence of co-crystallized solvent in the other series. However, [Au(S_2_C_2_(C_6_H_4_-4-^*t*^Bu)_2_)_2_]·CH_2_Cl_2_ crystallizes in *P*4_1_2_1_2 with unit cell parameters nearly identical to those of **1**·0.25(C_5_H_12_). Nickel–sulfur bond lengths generally assemble tightly at 2.12 Å. Although the resolution for its structure is somewhat more coarse, [Au(S_2_C_2_(C_6_H_4_-4-^*t*^Bu)_2_)_2_] differs from the Group 10 metal complexes in having, effectively, its di­thiol­ene ligand set halfway between redox states **a** and **b** in Fig. 2 ▸ such that the Au^3+^ ion is paired with three anionic ligand charges arising from one fully reduced dithiolate ligand and one half-oxidized monoanionic ligand. Consequently, its S—C and C—C_chelate_ bond lengths are longer and shorter, respectively, than those in its Group 10 counterparts. Conspicuous among the φ values for these compounds is the relatively large ≃ 66° angle observed for one unique Ph group in the [*M*(S_2_C_2_Ph_2_)_2_] (*M* = Ni, Pd, Pt) series, which has its origin in specific inter­molecular phenyl C—H⋯arene_centroid_ inter­actions that are not pertinent to **1**.

## 5. Synthesis and crystallization

**[Ni(S_2_C_2_(C_6_H_4_-4-** ***^t^*** **Bu)_2_)_2_], 1.** A mixture of 4,4′-di-*tert*-butyl­benzoin (0.350 g, 1.1 mmol) and P_4_S_10_ (0.355 g, 0.8 mmol) and dioxane (30 ml) in an oven-dried 100 ml three-neck flask was refluxed at 378 K for 12 h under N_2_ with continuous stirring. The reaction mixture was cooled to ambient temperature and then gravity filtered through paper in the open air into a 100 ml Schlenk flask. Nickel(II) dichloride hexa­hydrate (0.120 g, 0.5 mmol) dissolved in 1 ml of H_2_O was added to the filtrate, and reflux under N_2_ was recommenced and continued for 12 h with constant stirring. After being cooled to ambient temperature, the solid precipitate that formed was collected by vacuum filtration and then washed with CH_3_OH followed by Et_2_O. Yield: 0.135 g, 0.176 mmol, 32%. ^1^H NMR (δ, CDCl_3_): 7.33 (pseudo quartet, 16 H, aromatic C–H), 1.32 (*s*, 36 H, ^*t*^Bu). Analysis calculated for C_44_H_52_S_4_Ni: C, 68.83; H, 6.83; S, 16.70. Found: C, 68.71; H, 6.80; S, 16.63. This analysis was performed upon crystalline **1** grown by vapor diffusion of MeOH into a toluene solution, which produced crystals without inter­stitial solvent.

Vapor-diffusion methods were effective in generating crystals of diffraction quality. Crystals grown without inter­stitial solvent were complicated by significant non-merohedral twinning. However, introduction of *n*-pentane vapor into a THF solution of **1** produced crystalline **1**·0.25(C_5_H_12_) that was not subject to this problem or otherwise necessitating special treatment.

## 6. Refinement

Crystal data, data collection and structure refinement details are summarized in Table 2 ▸. The *tert*-butyl groups defined by C11–C14 and C41–C44 were disordered and treated with independent, floating site occupancy variables that identified 54:46 and 52:48 optimal partitioning, respectively, for the two groups. Hydrogen atoms were added in calculated positions and refined with isotropic displacement parameters that were approximately 1.2 times (for aromatic C—H) or 1.5 times (for –CH_3_) those of the carbon atoms to which they were attached. The C—H distances assumed were 0.95 and 0.98 Å for the aromatic C—H and –CH_3_ types of hydrogen atoms, respectively.

## Supplementary Material

Crystal structure: contains datablock(s) I, global. DOI: 10.1107/S205698902300097X/jy2027sup1.cif

Structure factors: contains datablock(s) I. DOI: 10.1107/S205698902300097X/jy2027Isup2.hkl

Supporting information file. DOI: 10.1107/S205698902300097X/jy2027sup3.txt

CCDC reference: 2239611

Additional supporting information:  crystallographic information; 3D view; checkCIF report

## Figures and Tables

**Table 1:** Structural parameters (Å, °) for selected [*M*(S_2_C_2_Ar_2_)_2_] complexes (*M* = Ni^2+^, Pd^2+^, Pt^2+^, Au^3+^; Ar = aryl group) φ represents the angles between the *M*S_4_C_4_ mean plane and the aryl C_6_ planes. Values of φ that were refined in *SHELXL* carry an uncertainty. All other values of φ were evaluated using *Mercury 3.7.*

Ar, *M*	Space group	*M*—S	S—C	C—C_chelate_	φ	Refcode
Ph, Ni^2+^	*P* 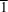	2.120, 2.127	1.701 (4), 1.695 (4)	1.424	50.64, 44.79	NIDPDS01^*a*^
		2.125, 2.125	1.718 (4), 1.702 (4)	1.404	53.06, 34.75	
Ph, Ni^2+^	*P*2_1_/*n*	2.1209 (6)	1.7152 (17)	1.388 (2)	34.20	NIDPDS03^*b*^
		2.1226 (7)	1.7035 (17)		65.77	
Ph, Pd^2+^	*P*2_1_/*n*	2.2502, 2.2496	1.696 (2), 1.712 (2)	1.399 (3)	35.83, 66.40	GOLRAA^*c*^
Ph, Pt^2+^	*P*2_1_/*n*	2.2443, 2.2460	1.6978, 1.7161	1.3965	35.87, 66.68	BUGDUC^*d*^
MeO-*p*-C_6_H_4_, Ni^2+^	*P* 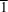	2.1221 (6)	1.7169 (19)		29.40	SICWO*R* ^*e*^
		2.1218 (6)	1.7029 (19)	1.393 (3)	53.00	
		2.1341 (5)	1.7171 (19)	1.391 (3)	41.61	
		2.1182 (6)	1.7100 (19)		39.91	
MeO-*p*-C_6_H_4_, Pd^2+^	*P* 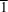	2.2535 (18)	1.699 (6)		40.24	SONPUI^*f*^
		2.2566 (18)	1.715 (6)	1.417 (9)	43.57	
		2.2706 (18)	1.711 (6)	1.411 (9)	30.22	
		2.2505 (18)	1.708 (6)		51.76	
MeO-*p*-C_6_H_4_, Pt^2+^	*P* 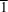	2.240 (2), 2.243 (2)	1.696 (9), 1.710 (7)	1.402 (12)	40.83, 42.04	SOPMOB^*f*^
		2.245 (3), 2.249 (3)	1.712 (8), 1.709 (9)	1.391 (12)	45.82, 38.35	
MeO-*p*-C_6_H_4_, Ni^2+^	*P* 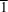	2.104 (3), 2.108 (3)	1.689 (5), 1.699 (5)	1.394 (6)	40.41, 43.96	ECEKAA^*b*^
		2.103 (3), 2.106 (3)	1.682 (5), 1.698 (5)	1.386 (6)	54.91, 35.28	
Cl-*p*-C_6_H_4_, Ni^2+^	*P* 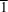	2.1277 (7)	1.706 (2)		35.39 (9)	DATTU*R* ^*g*^
		2.1192 (6)	1.704 (2)	1.399 (3)	54.34 (5)	
		2.1207 (7)	1.706 (2)	1.391 (3)	40.05 (6)	
		2.1261 (6)	1.713 (2)		42.99 (7)	
3,5-(MeO)_2_-4-BuO-C_6_H_2_, Ni^2+^	*P*2_1_/*n*	2.112	1.67 (1)		45.55	JUHJU*R* ^*h*^
^*t*^Bu-*p*-C_6_H_4_, Ni^2+^, **1**	*P*4_1_2_1_2	2.1175 (11)	1.717 (4)		41.7 (1)	This work
		2.1206 (12)	1.705 (4)	1.393 (6)	53.5 (1)	
		2.1280 (11)	1.705 (4)	1.403 (6)	53.4 (1)	
		2.1185 (11)	1.709 (4)		44.5 (2)	
^*t*^Bu-*p*-C_6_H_4_, Pd^2+^	*Pna*2_1_	2.2503 (10)			44.16	TEYSEW^*i*^
		2.2443 (10)	1.707 (4)	1.393 (5)	49.40	
		2.2667 (10)	1.712 (4)		51.77	
		2.2440 (10)			51.38	
^*t*^Bu-*p*-C_6_H_4_, Pt^2+^	*Pna*2_1_	2.243 (2), 2.242 (2)	1.728 (9), 1.729 (8)	1.381 (12)	48.37, 44.80	TIDBEO^*j*^
		2.259 (2), 2.243 (2)	1.709 (10), 1.685 (9)	1.404 (13)	52.30, 52.63	
^*t*^Bu-*p*-C_6_H_4_, Au^3+^	*P*4_1_2_1_2	2.284 (5), 2.288 (5)	1.74 (2), 1.745 (18)	1.38 (2)	41.16, 56.21	TEYSAS^*i*^
		2.290 (5), 2.303 (5)	1.751 (19). 1.76 (2)	1.33 (2)	47.30, 54.24	

Notes: (*a*) Megnamisi-Belombe & Nuber (1989 ▸); (*b*) Miao *et al.* (2011 ▸); (*c*) Sheu & Lee (1999 ▸); (*d*) Dessy *et al.* (1982 ▸); (*e*) Arumugam *et al.* (2007 ▸); (*f*) Chandrasekaran *et al.* (2014 ▸); (*g*) Koehne *et al.* (2022 ▸); (*h*) Nakazumi *et al.* (1992 ▸); (*i*) Kokatam *et al.* (2007 ▸); (*j*) Pap *et al.* (2007 ▸).

**Table 2:** Experimental details

Crystal data	
Chemical formula	[Ni(C_22_H_26_S_2_)_2_]·0.25C_5_H_12_
*M*_r_	785.84
Crystal system, space group	Tetragonal, *P*4_1_2_1_2
Temperature (K)	150
*a*, *c* (Å)	11.7187 (4), 65.014 (4)
*V* (Å^3^)	8928.2 (8)
*Z*	8
Radiation type	Cu *K*α
μ (mm^−1^)	2.58
Crystal size (mm)	0.21 × 0.11 × 0.05

Data collection	
Diffractometer	Bruker D8 VENTURE PHOTON 3 CPAD
Absorption correction	Multi-scan (*SADABS*; Krause *et al.*, 2015 ▸)
*T*_min_, *T*_max_	0.77, 0.88
No. of measured, independent and observed [*I* > 2σ(*I*)] reflections	203444, 8886, 8592
*R*_int_	0.072
(sin θ/λ)_max_ (Å^−1^)	0.619

Refinement	
*R*[*F* ^2^ > 2σ(*F* ^2^)], *wR*(*F* ^2^), *S*	0.045, 0.138, 1.06
No. of reflections	8886
No. of parameters	497
No. of restraints	27
H-atom treatment	H-atom parameters constrained
Δρ_max_, Δρ_min_ (e Å^−3^)	1.57, −0.40
Absolute structure	Refined as an inversion twin.
Absolute structure parameter	0.03 (2)

Computer programs: *APEX4* and *SAINT* (Bruker, 2021 ▸), *SHELXT* (Sheldrick, 2015*a* ▸), *SHELXL2018/1* (Sheldrick, 2015*b* ▸), and *SHELXTL* (Sheldrick, 2008 ▸).

## supplementary crystallographic information

### Crystal data

**Table:**

[Ni(C_22_H_26_S_2_)_2_]·0.25C_5_H_12_	*D*_x_ = 1.169 Mg m^−^^3^
*M**_r_* = 785.84	Cu *K*α radiation, λ = 1.54178 Å
Tetragonal, *P*4_1_2_1_2	Cell parameters from 9381 reflections
*a* = 11.7187 (4) Å	θ = 4.0–72.6°
*c* = 65.014 (4) Å	µ = 2.58 mm^−^^1^
*V* = 8928.2 (8) Å^3^	*T* = 150 K
*Z* = 8	Column, black
*F*(000) = 3348	0.21 × 0.11 × 0.05 mm

### Data collection

**Table:**

Bruker D8 VENTURE PHOTON 3 CPAD diffractometer	8886 independent reflections
Radiation source: INCOATEC IµS micro—-focus source	8592 reflections with *I* > 2σ(*I*)
Mirror monochromator	*R*_int_ = 0.072
Detector resolution: 7.3910 pixels mm^-1^	θ_max_ = 72.7°, θ_min_ = 4.0°
φ and ω scans	*h* = −14→14
Absorption correction: multi-scan (*SADABS*; Krause *et al.*, 2015)	*k* = −14→14
*T*_min_ = 0.77, *T*_max_ = 0.88	*l* = −80→80
203444 measured reflections	

### Refinement

**Table:**

Refinement on *F*^2^	Secondary atom site location: difference Fourier map
Least-squares matrix: full	Hydrogen site location: inferred from neighbouring sites
*R*[*F*^2^ > 2σ(*F*^2^)] = 0.045	H-atom parameters constrained
*wR*(*F*^2^) = 0.138	*w* = 1/[σ^2^(*F*_o_^2^) + (0.0951*P*)^2^ + 5.7915*P*] where *P* = (*F*_o_^2^ + 2*F*_c_^2^)/3
*S* = 1.06	(Δ/σ)_max_ = 0.003
8886 reflections	Δρ_max_ = 1.57 e Å^−^^3^
497 parameters	Δρ_min_ = −0.40 e Å^−^^3^
27 restraints	Absolute structure: Refined as an inversion twin.
Primary atom site location: dual	Absolute structure parameter: 0.03 (2)

### Special details

**Table:**

Geometry. All esds (except the esd in the dihedral angle between two l.s. planes) are estimated using the full covariance matrix. The cell esds are taken into account individually in the estimation of esds in distances, angles and torsion angles; correlations between esds in cell parameters are only used when they are defined by crystal symmetry. An approximate (isotropic) treatment of cell esds is used for estimating esds involving l.s. planes.
Refinement. Refined as a 2-component inversion twin.

### Fractional atomic coordinates and isotropic or equivalent isotropic displacement parameters (Å^2^)

**Table:**

	*x*	*y*	*z*	*U*_iso_*/*U*_eq_	Occ. (<1)
Ni1	0.11325 (6)	0.50471 (6)	0.66096 (2)	0.02572 (18)	
S1	0.16585 (8)	0.54152 (9)	0.63052 (2)	0.0270 (2)	
S2	0.26404 (8)	0.40913 (9)	0.66631 (2)	0.0284 (2)	
S3	−0.03961 (8)	0.59838 (8)	0.65575 (2)	0.0264 (2)	
S4	0.06448 (9)	0.46883 (9)	0.69166 (2)	0.0318 (2)	
C1	0.2970 (3)	0.4783 (3)	0.62760 (6)	0.0252 (7)	
C2	0.3415 (3)	0.4174 (3)	0.64413 (6)	0.0253 (7)	
C3	−0.1135 (3)	0.5915 (3)	0.67829 (6)	0.0269 (8)	
C4	−0.0659 (3)	0.5317 (3)	0.69492 (6)	0.0295 (8)	
C5	0.3489 (3)	0.4871 (3)	0.60685 (6)	0.0244 (7)	
C6	0.3989 (3)	0.3928 (4)	0.59745 (6)	0.0277 (8)	
H6	0.408370	0.323934	0.604960	0.033*	
C7	0.4348 (3)	0.3990 (4)	0.57716 (6)	0.0300 (8)	
H7	0.467590	0.333322	0.570971	0.036*	
C8	0.4244 (3)	0.4987 (4)	0.56554 (6)	0.0303 (8)	
C9	0.3773 (4)	0.5935 (4)	0.57528 (6)	0.0308 (8)	
H9	0.370156	0.662995	0.567872	0.037*	
C10	0.3406 (3)	0.5888 (4)	0.59557 (6)	0.0271 (8)	
H10	0.309538	0.655085	0.601869	0.032*	
C11	0.4587 (4)	0.5019 (5)	0.54280 (7)	0.0394 (10)	
C12A	0.3892 (19)	0.4182 (18)	0.5312 (2)	0.074 (7)	0.461 (19)
H12A	0.421610	0.407950	0.517424	0.111*	0.461 (19)
H12B	0.310697	0.446374	0.530004	0.111*	0.461 (19)
H12C	0.389198	0.344980	0.538470	0.111*	0.461 (19)
C13A	0.4460 (16)	0.6250 (14)	0.53373 (19)	0.058 (4)	0.461 (19)
H13A	0.487709	0.629958	0.520695	0.087*	0.461 (19)
H13B	0.477300	0.680560	0.543481	0.087*	0.461 (19)
H13C	0.365115	0.641493	0.531339	0.087*	0.461 (19)
C14A	0.5838 (13)	0.4692 (18)	0.54094 (19)	0.068 (6)	0.461 (19)
H14A	0.593129	0.414042	0.529742	0.102*	0.461 (19)
H14B	0.609666	0.435055	0.553881	0.102*	0.461 (19)
H14C	0.629133	0.537554	0.537998	0.102*	0.461 (19)
C12B	0.3464 (13)	0.500 (2)	0.5301 (2)	0.086 (6)	0.539 (19)
H12D	0.311742	0.576312	0.530291	0.129*	0.539 (19)
H12E	0.293295	0.444965	0.536151	0.129*	0.539 (19)
H12F	0.363294	0.478302	0.515887	0.129*	0.539 (19)
C13B	0.530 (2)	0.6003 (19)	0.5382 (2)	0.106 (10)	0.539 (19)
H13D	0.492102	0.669788	0.543104	0.158*	0.539 (19)
H13E	0.541539	0.605513	0.523298	0.158*	0.539 (19)
H13F	0.603756	0.591920	0.545073	0.158*	0.539 (19)
C14B	0.510 (2)	0.3851 (15)	0.5356 (2)	0.097 (8)	0.539 (19)
H14D	0.536602	0.391757	0.521379	0.146*	0.539 (19)
H14E	0.451461	0.325667	0.536495	0.146*	0.539 (19)
H14F	0.574518	0.364714	0.544519	0.146*	0.539 (19)
C15	0.4557 (3)	0.3629 (3)	0.64462 (6)	0.0247 (7)	
C16	0.4669 (3)	0.2503 (3)	0.65136 (6)	0.0257 (7)	
H16	0.400958	0.207967	0.655083	0.031*	
C17	0.5738 (3)	0.1998 (3)	0.65265 (6)	0.0267 (7)	
H17	0.579397	0.122266	0.656774	0.032*	
C18	0.6728 (3)	0.2598 (3)	0.64807 (6)	0.0254 (7)	
C19	0.6602 (3)	0.3733 (4)	0.64133 (7)	0.0329 (9)	
H19	0.726089	0.416447	0.637899	0.039*	
C20	0.5537 (3)	0.4231 (4)	0.63961 (7)	0.0331 (9)	
H20	0.547600	0.499669	0.634928	0.040*	
C21	0.7914 (3)	0.2093 (3)	0.65087 (6)	0.0289 (8)	
C22	0.8510 (4)	0.2736 (4)	0.66836 (8)	0.0411 (10)	
H22A	0.856736	0.354653	0.664813	0.062*	
H22B	0.927732	0.242173	0.670390	0.062*	
H22C	0.806794	0.265018	0.681056	0.062*	
C23	0.7887 (4)	0.0820 (4)	0.65608 (8)	0.0383 (10)	
H23A	0.753235	0.071129	0.669589	0.057*	
H23B	0.866802	0.052053	0.656359	0.057*	
H23C	0.744385	0.041142	0.645628	0.057*	
C24	0.8616 (4)	0.2242 (5)	0.63087 (9)	0.0492 (13)	
H24A	0.816219	0.198526	0.619089	0.074*	
H24B	0.931582	0.178616	0.631763	0.074*	
H24C	0.881503	0.304810	0.629110	0.074*	
C25	−0.2282 (3)	0.6431 (3)	0.67841 (6)	0.0267 (8)	
C26	−0.3229 (4)	0.5781 (4)	0.68385 (9)	0.0425 (11)	
H26	−0.313031	0.501166	0.688097	0.051*	
C27	−0.4317 (4)	0.6248 (4)	0.68311 (9)	0.0453 (12)	
H27	−0.495056	0.578704	0.686895	0.054*	
C28	−0.4510 (3)	0.7372 (4)	0.67700 (6)	0.0291 (8)	
C29	−0.3557 (4)	0.8014 (4)	0.67168 (7)	0.0319 (8)	
H29	−0.365326	0.878569	0.667577	0.038*	
C30	−0.2463 (4)	0.7548 (4)	0.67226 (7)	0.0319 (9)	
H30	−0.182968	0.800535	0.668360	0.038*	
C31	−0.5721 (4)	0.7830 (4)	0.67471 (7)	0.0347 (9)	
C32	−0.5777 (5)	0.9115 (5)	0.67685 (13)	0.0668 (19)	
H32A	−0.542830	0.934267	0.689922	0.100*	
H32B	−0.657541	0.936162	0.676570	0.100*	
H32C	−0.536216	0.947115	0.665444	0.100*	
C33	−0.6538 (5)	0.7297 (6)	0.69042 (11)	0.0655 (18)	
H33A	−0.620952	0.736244	0.704226	0.098*	
H33B	−0.665624	0.649050	0.687078	0.098*	
H33C	−0.727136	0.769902	0.689986	0.098*	
C34	−0.6154 (5)	0.7513 (6)	0.65312 (9)	0.0584 (15)	
H34A	−0.698181	0.762907	0.652457	0.088*	
H34B	−0.597801	0.671104	0.650284	0.088*	
H34C	−0.577955	0.799788	0.642861	0.088*	
C35	−0.1179 (4)	0.5215 (4)	0.71562 (6)	0.0331 (8)	
C36	−0.1179 (5)	0.4183 (4)	0.72576 (7)	0.0448 (11)	
H36	−0.088892	0.352356	0.719052	0.054*	
C37	−0.1602 (5)	0.4093 (5)	0.74586 (7)	0.0480 (12)	
H37	−0.159235	0.337265	0.752549	0.058*	
C38	−0.2035 (5)	0.5031 (5)	0.75617 (7)	0.0464 (12)	
C39	−0.2077 (6)	0.6049 (5)	0.74547 (8)	0.0572 (15)	
H39	−0.239496	0.669983	0.752028	0.069*	
C40	−0.1672 (5)	0.6156 (4)	0.72546 (8)	0.0494 (13)	
H40	−0.173016	0.686603	0.718481	0.059*	
C41	−0.2483 (5)	0.4953 (5)	0.77823 (7)	0.0585 (16)	
C42A	−0.1891 (16)	0.3937 (11)	0.78986 (19)	0.051 (4)*	0.48 (3)
H42A	−0.218619	0.389213	0.803941	0.077*	0.48 (3)
H42B	−0.106439	0.406361	0.790249	0.077*	0.48 (3)
H42C	−0.205229	0.322032	0.782647	0.077*	0.48 (3)
C43A	−0.227 (2)	0.6045 (12)	0.7902 (2)	0.059 (4)*	0.48 (3)
H43A	−0.267924	0.667602	0.783673	0.089*	0.48 (3)
H43B	−0.144626	0.621226	0.790162	0.089*	0.48 (3)
H43C	−0.253082	0.595077	0.804358	0.089*	0.48 (3)
C44A	−0.3728 (12)	0.465 (3)	0.7777 (3)	0.083 (6)*	0.48 (3)
H44A	−0.402360	0.461900	0.791837	0.124*	0.48 (3)
H44B	−0.382656	0.390692	0.771135	0.124*	0.48 (3)
H44C	−0.414595	0.523307	0.769961	0.124*	0.48 (3)
C42B	−0.2387 (19)	0.3819 (9)	0.7882 (2)	0.061 (4)*	0.52 (3)
H42D	−0.284956	0.326412	0.780606	0.092*	0.52 (3)
H42E	−0.266099	0.386955	0.802407	0.092*	0.52 (3)
H42F	−0.158737	0.357487	0.788152	0.092*	0.52 (3)
C43B	−0.1921 (14)	0.5929 (10)	0.79087 (17)	0.044 (3)*	0.52 (3)
H43D	−0.210581	0.583221	0.805470	0.066*	0.52 (3)
H43E	−0.221200	0.666640	0.786075	0.066*	0.52 (3)
H43F	−0.109150	0.590326	0.789038	0.066*	0.52 (3)
C44B	−0.3796 (9)	0.5117 (18)	0.7777 (2)	0.064 (4)*	0.52 (3)
H44D	−0.414406	0.449483	0.769802	0.096*	0.52 (3)
H44E	−0.397658	0.584817	0.771121	0.096*	0.52 (3)
H44F	−0.409572	0.511207	0.791743	0.096*	0.52 (3)
C45	−0.6695 (19)	0.6695 (19)	0.750000	0.104 (9)*	0.5
H45A	−0.625613	0.640961	0.738040	0.125*	0.25
H45B	−0.640954	0.625607	0.761958	0.125*	0.25
C46	−0.621 (2)	0.796 (2)	0.7537 (4)	0.126 (8)*	0.5
H46A	−0.665818	0.849448	0.745177	0.152*	0.5
H46B	−0.633358	0.816350	0.768245	0.152*	0.5
C47	−0.497 (2)	0.814 (2)	0.7489 (4)	0.138 (9)*	0.5
H47A	−0.476819	0.893635	0.751644	0.208*	0.5
H47B	−0.450667	0.763562	0.757474	0.208*	0.5
H47C	−0.483233	0.796770	0.734331	0.208*	0.5

### Atomic displacement parameters (Å^2^)

**Table:**

	*U*^11^	*U*^22^	*U*^33^	*U*^12^	*U*^13^	*U*^23^
Ni1	0.0209 (3)	0.0316 (4)	0.0247 (3)	0.0036 (2)	0.0032 (2)	0.0019 (3)
S1	0.0211 (4)	0.0343 (5)	0.0257 (4)	0.0055 (3)	0.0021 (3)	0.0047 (4)
S2	0.0234 (4)	0.0357 (5)	0.0263 (4)	0.0056 (4)	0.0023 (3)	0.0051 (4)
S3	0.0229 (4)	0.0314 (5)	0.0249 (4)	0.0042 (3)	0.0037 (3)	0.0034 (3)
S4	0.0290 (5)	0.0412 (5)	0.0252 (4)	0.0091 (4)	0.0026 (3)	0.0044 (4)
C1	0.0194 (16)	0.0253 (17)	0.0309 (18)	−0.0014 (14)	0.0005 (14)	−0.0002 (15)
C2	0.0218 (17)	0.0276 (18)	0.0266 (17)	−0.0019 (14)	0.0007 (14)	−0.0009 (15)
C3	0.0256 (18)	0.0277 (19)	0.0275 (18)	−0.0003 (15)	0.0060 (15)	−0.0006 (15)
C4	0.0286 (18)	0.0312 (19)	0.0287 (18)	0.0025 (16)	0.0030 (15)	0.0009 (15)
C5	0.0164 (15)	0.0282 (19)	0.0286 (17)	−0.0003 (13)	−0.0017 (13)	0.0029 (15)
C6	0.0214 (17)	0.0296 (19)	0.0320 (19)	0.0018 (15)	0.0000 (15)	0.0013 (15)
C7	0.0244 (18)	0.034 (2)	0.032 (2)	0.0011 (15)	0.0011 (15)	−0.0030 (16)
C8	0.0217 (18)	0.042 (2)	0.0275 (19)	−0.0056 (16)	0.0012 (15)	−0.0005 (17)
C9	0.0264 (19)	0.035 (2)	0.031 (2)	−0.0045 (16)	−0.0010 (15)	0.0049 (16)
C10	0.0215 (17)	0.0301 (19)	0.0295 (19)	−0.0002 (15)	−0.0007 (14)	0.0033 (16)
C11	0.041 (2)	0.052 (3)	0.0252 (19)	−0.005 (2)	0.0031 (18)	−0.0001 (19)
C12A	0.095 (15)	0.097 (14)	0.029 (6)	−0.052 (12)	0.006 (7)	−0.005 (7)
C13A	0.072 (10)	0.074 (9)	0.028 (5)	0.007 (8)	0.012 (6)	0.019 (5)
C14A	0.061 (8)	0.111 (15)	0.032 (6)	0.024 (9)	0.031 (6)	0.017 (7)
C12B	0.064 (8)	0.16 (2)	0.037 (6)	0.010 (10)	−0.011 (5)	−0.001 (9)
C13B	0.133 (19)	0.132 (17)	0.052 (8)	−0.075 (16)	0.049 (11)	−0.023 (9)
C14B	0.16 (2)	0.083 (11)	0.052 (7)	0.056 (13)	0.039 (10)	−0.007 (7)
C15	0.0208 (17)	0.0276 (18)	0.0257 (17)	0.0022 (14)	−0.0011 (14)	0.0024 (14)
C16	0.0232 (17)	0.0265 (18)	0.0274 (17)	−0.0030 (14)	−0.0003 (14)	−0.0008 (15)
C17	0.0266 (18)	0.0231 (17)	0.0304 (18)	−0.0003 (14)	−0.0009 (15)	0.0011 (15)
C18	0.0214 (17)	0.0282 (19)	0.0266 (17)	0.0034 (15)	−0.0009 (14)	0.0000 (14)
C19	0.0205 (18)	0.031 (2)	0.048 (2)	0.0012 (15)	0.0031 (17)	0.0103 (18)
C20	0.0236 (19)	0.0287 (19)	0.047 (2)	0.0016 (16)	0.0011 (17)	0.0137 (18)
C21	0.0240 (18)	0.030 (2)	0.033 (2)	0.0049 (15)	0.0013 (15)	0.0011 (16)
C22	0.026 (2)	0.042 (2)	0.055 (3)	0.0059 (18)	−0.0105 (19)	−0.004 (2)
C23	0.030 (2)	0.030 (2)	0.055 (3)	0.0086 (17)	−0.0027 (19)	0.0017 (19)
C24	0.035 (2)	0.060 (3)	0.053 (3)	0.017 (2)	0.017 (2)	0.011 (2)
C25	0.0255 (19)	0.0282 (19)	0.0264 (17)	0.0009 (15)	0.0043 (14)	−0.0009 (15)
C26	0.030 (2)	0.032 (2)	0.066 (3)	0.0067 (18)	0.016 (2)	0.013 (2)
C27	0.028 (2)	0.030 (2)	0.078 (4)	0.0019 (17)	0.019 (2)	0.013 (2)
C28	0.0240 (18)	0.030 (2)	0.0337 (19)	0.0036 (15)	0.0044 (15)	−0.0020 (16)
C29	0.027 (2)	0.0261 (19)	0.042 (2)	0.0004 (15)	0.0012 (17)	0.0051 (17)
C30	0.027 (2)	0.030 (2)	0.039 (2)	−0.0010 (16)	0.0019 (16)	0.0042 (17)
C31	0.029 (2)	0.031 (2)	0.045 (2)	0.0054 (16)	0.0057 (17)	0.0025 (17)
C32	0.034 (3)	0.034 (3)	0.133 (6)	0.013 (2)	−0.009 (3)	−0.015 (3)
C33	0.033 (3)	0.078 (4)	0.086 (4)	0.019 (3)	0.025 (3)	0.022 (4)
C34	0.036 (3)	0.077 (4)	0.062 (3)	0.023 (3)	−0.009 (2)	−0.018 (3)
C35	0.032 (2)	0.039 (2)	0.0279 (19)	0.0029 (17)	0.0062 (16)	0.0016 (16)
C36	0.062 (3)	0.039 (2)	0.034 (2)	0.009 (2)	0.014 (2)	0.0048 (19)
C37	0.070 (4)	0.042 (3)	0.031 (2)	0.003 (2)	0.013 (2)	0.007 (2)
C38	0.063 (3)	0.047 (3)	0.029 (2)	−0.006 (2)	0.013 (2)	0.000 (2)
C39	0.085 (4)	0.046 (3)	0.041 (3)	0.006 (3)	0.024 (3)	−0.008 (2)
C40	0.075 (4)	0.038 (2)	0.036 (2)	0.008 (2)	0.019 (2)	0.006 (2)
C41	0.087 (4)	0.056 (3)	0.032 (2)	−0.011 (3)	0.023 (3)	−0.002 (2)

### Geometric parameters (Å, º)

**Table:**

Ni1—S1	2.1174 (11)	C24—H24B	0.9800
Ni1—S4	2.1185 (11)	C24—H24C	0.9800
Ni1—S2	2.1207 (11)	C25—C30	1.386 (6)
Ni1—S3	2.1281 (11)	C25—C26	1.392 (6)
S1—C1	1.716 (4)	C26—C27	1.389 (6)
S2—C2	1.707 (4)	C26—H26	0.9500
S3—C3	1.704 (4)	C27—C28	1.394 (6)
S4—C4	1.710 (4)	C27—H27	0.9500
C1—C2	1.392 (5)	C28—C29	1.390 (6)
C1—C5	1.484 (5)	C28—C31	1.525 (6)
C2—C15	1.483 (5)	C29—C30	1.394 (6)
C3—C4	1.404 (6)	C29—H29	0.9500
C3—C25	1.473 (5)	C30—H30	0.9500
C4—C35	1.482 (5)	C31—C32	1.513 (7)
C5—C6	1.392 (6)	C31—C33	1.533 (7)
C5—C10	1.402 (5)	C31—C34	1.538 (7)
C6—C7	1.386 (6)	C32—H32A	0.9800
C6—H6	0.9500	C32—H32B	0.9800
C7—C8	1.396 (6)	C32—H32C	0.9800
C7—H7	0.9500	C33—H33A	0.9800
C8—C9	1.393 (6)	C33—H33B	0.9800
C8—C11	1.532 (5)	C33—H33C	0.9800
C9—C10	1.389 (6)	C34—H34A	0.9800
C9—H9	0.9500	C34—H34B	0.9800
C10—H10	0.9500	C34—H34C	0.9800
C11—C13B	1.455 (16)	C35—C36	1.378 (7)
C11—C12A	1.481 (15)	C35—C40	1.400 (7)
C11—C14A	1.520 (14)	C36—C37	1.402 (6)
C11—C12B	1.553 (14)	C36—H36	0.9500
C11—C13A	1.566 (15)	C37—C38	1.384 (8)
C11—C14B	1.567 (14)	C37—H37	0.9500
C12A—H12A	0.9800	C38—C39	1.382 (8)
C12A—H12B	0.9800	C38—C41	1.530 (6)
C12A—H12C	0.9800	C39—C40	1.391 (7)
C13A—H13A	0.9800	C39—H39	0.9500
C13A—H13B	0.9800	C40—H40	0.9500
C13A—H13C	0.9800	C41—C42B	1.482 (10)
C14A—H14A	0.9800	C41—C44A	1.501 (12)
C14A—H14B	0.9800	C41—C43A	1.518 (11)
C14A—H14C	0.9800	C41—C44B	1.551 (11)
C12B—H12D	0.9800	C41—C43B	1.554 (10)
C12B—H12E	0.9800	C41—C42A	1.572 (10)
C12B—H12F	0.9800	C42A—H42A	0.9800
C13B—H13D	0.9800	C42A—H42B	0.9800
C13B—H13E	0.9800	C42A—H42C	0.9800
C13B—H13F	0.9800	C43A—H43A	0.9800
C14B—H14D	0.9800	C43A—H43B	0.9800
C14B—H14E	0.9800	C43A—H43C	0.9800
C14B—H14F	0.9800	C44A—H44A	0.9800
C15—C20	1.387 (6)	C44A—H44B	0.9800
C15—C16	1.397 (5)	C44A—H44C	0.9800
C16—C17	1.388 (6)	C42B—H42D	0.9800
C16—H16	0.9500	C42B—H42E	0.9800
C17—C18	1.389 (6)	C42B—H42F	0.9800
C17—H17	0.9500	C43B—H43D	0.9800
C18—C19	1.409 (6)	C43B—H43E	0.9800
C18—C21	1.521 (5)	C43B—H43F	0.9800
C19—C20	1.382 (6)	C44B—H44D	0.9800
C19—H19	0.9500	C44B—H44E	0.9800
C20—H20	0.9500	C44B—H44F	0.9800
C21—C23	1.530 (6)	C45—C46^i^	1.61 (3)
C21—C22	1.533 (6)	C45—C46	1.61 (3)
C21—C24	1.548 (6)	C45—H45A	0.9900
C22—H22A	0.9800	C45—H45B	0.9900
C22—H22B	0.9800	C46—C47	1.49 (3)
C22—H22C	0.9800	C46—H46A	0.9900
C23—H23A	0.9800	C46—H46B	0.9900
C23—H23B	0.9800	C47—H47A	0.9800
C23—H23C	0.9800	C47—H47B	0.9800
C24—H24A	0.9800	C47—H47C	0.9800
			
S1—Ni1—S4	178.67 (5)	H24A—C24—H24C	109.5
S1—Ni1—S2	91.05 (4)	H24B—C24—H24C	109.5
S4—Ni1—S2	88.02 (4)	C30—C25—C26	117.9 (4)
S1—Ni1—S3	89.49 (4)	C30—C25—C3	121.7 (4)
S4—Ni1—S3	91.45 (4)	C26—C25—C3	120.3 (4)
S2—Ni1—S3	179.10 (6)	C27—C26—C25	120.6 (4)
C1—S1—Ni1	106.01 (14)	C27—C26—H26	119.7
C2—S2—Ni1	105.95 (14)	C25—C26—H26	119.7
C3—S3—Ni1	105.44 (14)	C26—C27—C28	122.1 (4)
C4—S4—Ni1	105.80 (14)	C26—C27—H27	119.0
C2—C1—C5	125.8 (3)	C28—C27—H27	119.0
C2—C1—S1	118.2 (3)	C29—C28—C27	116.9 (4)
C5—C1—S1	115.9 (3)	C29—C28—C31	122.2 (4)
C1—C2—C15	125.2 (3)	C27—C28—C31	120.7 (4)
C1—C2—S2	118.8 (3)	C28—C29—C30	121.4 (4)
C15—C2—S2	115.9 (3)	C28—C29—H29	119.3
C4—C3—C25	124.3 (4)	C30—C29—H29	119.3
C4—C3—S3	119.0 (3)	C25—C30—C29	121.3 (4)
C25—C3—S3	116.6 (3)	C25—C30—H30	119.4
C3—C4—C35	125.2 (4)	C29—C30—H30	119.4
C3—C4—S4	118.3 (3)	C32—C31—C28	112.4 (4)
C35—C4—S4	116.4 (3)	C32—C31—C33	108.5 (5)
C6—C5—C10	118.3 (4)	C28—C31—C33	111.9 (4)
C6—C5—C1	121.1 (4)	C32—C31—C34	108.0 (5)
C10—C5—C1	120.4 (4)	C28—C31—C34	108.2 (4)
C7—C6—C5	120.2 (4)	C33—C31—C34	107.7 (5)
C7—C6—H6	119.9	C31—C32—H32A	109.5
C5—C6—H6	119.9	C31—C32—H32B	109.5
C6—C7—C8	122.2 (4)	H32A—C32—H32B	109.5
C6—C7—H7	118.9	C31—C32—H32C	109.5
C8—C7—H7	118.9	H32A—C32—H32C	109.5
C9—C8—C7	117.1 (4)	H32B—C32—H32C	109.5
C9—C8—C11	121.5 (4)	C31—C33—H33A	109.5
C7—C8—C11	121.3 (4)	C31—C33—H33B	109.5
C10—C9—C8	121.6 (4)	H33A—C33—H33B	109.5
C10—C9—H9	119.2	C31—C33—H33C	109.5
C8—C9—H9	119.2	H33A—C33—H33C	109.5
C9—C10—C5	120.6 (4)	H33B—C33—H33C	109.5
C9—C10—H10	119.7	C31—C34—H34A	109.5
C5—C10—H10	119.7	C31—C34—H34B	109.5
C12A—C11—C14A	108.9 (12)	H34A—C34—H34B	109.5
C13B—C11—C8	111.6 (7)	C31—C34—H34C	109.5
C12A—C11—C8	109.3 (6)	H34A—C34—H34C	109.5
C14A—C11—C8	108.9 (6)	H34B—C34—H34C	109.5
C13B—C11—C12B	112.7 (13)	C36—C35—C40	118.2 (4)
C8—C11—C12B	106.9 (6)	C36—C35—C4	120.3 (4)
C12A—C11—C13A	111.5 (11)	C40—C35—C4	121.4 (4)
C14A—C11—C13A	107.1 (10)	C35—C36—C37	120.8 (5)
C8—C11—C13A	111.2 (6)	C35—C36—H36	119.6
C13B—C11—C14B	114.3 (12)	C37—C36—H36	119.6
C8—C11—C14B	111.5 (6)	C38—C37—C36	121.5 (5)
C12B—C11—C14B	99.0 (12)	C38—C37—H37	119.3
C11—C12A—H12A	109.5	C36—C37—H37	119.3
C11—C12A—H12B	109.5	C39—C38—C37	117.0 (4)
H12A—C12A—H12B	109.5	C39—C38—C41	120.8 (5)
C11—C12A—H12C	109.5	C37—C38—C41	122.2 (5)
H12A—C12A—H12C	109.5	C38—C39—C40	122.5 (5)
H12B—C12A—H12C	109.5	C38—C39—H39	118.8
C11—C13A—H13A	109.5	C40—C39—H39	118.8
C11—C13A—H13B	109.5	C39—C40—C35	119.8 (5)
H13A—C13A—H13B	109.5	C39—C40—H40	120.1
C11—C13A—H13C	109.5	C35—C40—H40	120.1
H13A—C13A—H13C	109.5	C44A—C41—C43A	111.8 (12)
H13B—C13A—H13C	109.5	C42B—C41—C38	115.9 (7)
C11—C14A—H14A	109.5	C44A—C41—C38	109.1 (9)
C11—C14A—H14B	109.5	C43A—C41—C38	111.8 (8)
H14A—C14A—H14B	109.5	C42B—C41—C44B	101.3 (9)
C11—C14A—H14C	109.5	C38—C41—C44B	108.1 (7)
H14A—C14A—H14C	109.5	C42B—C41—C43B	113.4 (8)
H14B—C14A—H14C	109.5	C38—C41—C43B	107.8 (6)
C11—C12B—H12D	109.5	C44B—C41—C43B	110.0 (9)
C11—C12B—H12E	109.5	C44A—C41—C42A	105.1 (11)
H12D—C12B—H12E	109.5	C43A—C41—C42A	108.6 (9)
C11—C12B—H12F	109.5	C38—C41—C42A	110.2 (7)
H12D—C12B—H12F	109.5	C41—C42A—H42A	109.5
H12E—C12B—H12F	109.5	C41—C42A—H42B	109.5
C11—C13B—H13D	109.5	H42A—C42A—H42B	109.5
C11—C13B—H13E	109.5	C41—C42A—H42C	109.5
H13D—C13B—H13E	109.5	H42A—C42A—H42C	109.5
C11—C13B—H13F	109.5	H42B—C42A—H42C	109.5
H13D—C13B—H13F	109.5	C41—C43A—H43A	109.5
H13E—C13B—H13F	109.5	C41—C43A—H43B	109.5
C11—C14B—H14D	109.5	H43A—C43A—H43B	109.5
C11—C14B—H14E	109.5	C41—C43A—H43C	109.5
H14D—C14B—H14E	109.5	H43A—C43A—H43C	109.5
C11—C14B—H14F	109.5	H43B—C43A—H43C	109.5
H14D—C14B—H14F	109.5	C41—C44A—H44A	109.5
H14E—C14B—H14F	109.5	C41—C44A—H44B	109.5
C20—C15—C16	118.5 (4)	H44A—C44A—H44B	109.5
C20—C15—C2	121.5 (3)	C41—C44A—H44C	109.5
C16—C15—C2	119.9 (4)	H44A—C44A—H44C	109.5
C17—C16—C15	120.4 (4)	H44B—C44A—H44C	109.5
C17—C16—H16	119.8	C41—C42B—H42D	109.5
C15—C16—H16	119.8	C41—C42B—H42E	109.5
C16—C17—C18	121.7 (4)	H42D—C42B—H42E	109.5
C16—C17—H17	119.1	C41—C42B—H42F	109.5
C18—C17—H17	119.1	H42D—C42B—H42F	109.5
C17—C18—C19	117.1 (4)	H42E—C42B—H42F	109.5
C17—C18—C21	122.7 (4)	C41—C43B—H43D	109.5
C19—C18—C21	120.1 (4)	C41—C43B—H43E	109.5
C20—C19—C18	121.3 (4)	H43D—C43B—H43E	109.5
C20—C19—H19	119.3	C41—C43B—H43F	109.5
C18—C19—H19	119.3	H43D—C43B—H43F	109.5
C19—C20—C15	120.9 (4)	H43E—C43B—H43F	109.5
C19—C20—H20	119.6	C41—C44B—H44D	109.5
C15—C20—H20	119.6	C41—C44B—H44E	109.5
C18—C21—C23	112.8 (3)	H44D—C44B—H44E	109.5
C18—C21—C22	108.3 (3)	C41—C44B—H44F	109.5
C23—C21—C22	109.0 (4)	H44D—C44B—H44F	109.5
C18—C21—C24	109.9 (3)	H44E—C44B—H44F	109.5
C23—C21—C24	107.9 (4)	C46^i^—C45—C46	133 (3)
C22—C21—C24	109.0 (4)	C46^i^—C45—H45A	104.0
C21—C22—H22A	109.5	C46—C45—H45A	104.0
C21—C22—H22B	109.5	C46^i^—C45—H45B	104.0
H22A—C22—H22B	109.5	C46—C45—H45B	104.0
C21—C22—H22C	109.5	H45A—C45—H45B	105.5
H22A—C22—H22C	109.5	C47—C46—C45	116 (2)
H22B—C22—H22C	109.5	C47—C46—H46A	108.2
C21—C23—H23A	109.5	C45—C46—H46A	108.2
C21—C23—H23B	109.5	C47—C46—H46B	108.2
H23A—C23—H23B	109.5	C45—C46—H46B	108.2
C21—C23—H23C	109.5	H46A—C46—H46B	107.3
H23A—C23—H23C	109.5	C46—C47—H47A	109.5
H23B—C23—H23C	109.5	C46—C47—H47B	109.5
C21—C24—H24A	109.5	H47A—C47—H47B	109.5
C21—C24—H24B	109.5	C46—C47—H47C	109.5
H24A—C24—H24B	109.5	H47A—C47—H47C	109.5
C21—C24—H24C	109.5	H47B—C47—H47C	109.5

Symmetry code: (i) −*y*, −*x*, −*z*+3/2.
